# The Association of Prenatal Exposure to Perfluorinated Chemicals with Maternal Essential and Long-Chain Polyunsaturated Fatty Acids during Pregnancy and the Birth Weight of Their Offspring: The Hokkaido Study

**DOI:** 10.1289/ehp.1408834

**Published:** 2015-04-03

**Authors:** Reiko Kishi, Tamie Nakajima, Houman Goudarzi, Sachiko Kobayashi, Seiko Sasaki, Emiko Okada, Chihiro Miyashita, Sachiko Itoh, Atsuko Araki, Tamiko Ikeno, Yusuke Iwasaki, Hiroyuki Nakazawa

**Affiliations:** 1 Center for Environmental and Health Sciences, Hokkaido University, Sapporo, Japan; 2 College of Life and Health Sciences, Chubu University, Kasugai, Japan; 3 Department of Occupational and Environmental Health, Nagoya University Graduate School of Medicine, Nagoya, Japan; 4 Department of Public Health, Hokkaido University Graduate School of Medicine, Sapporo, Japan; 5 Department of Analytical Chemistry, Faculty of Pharmaceutical Sciences, Hoshi University, Tokyo, Japan

## Abstract

**Background:**

Fatty acids (FAs) are essential for fetal growth. Exposure to perfluorinated chemicals (PFCs) may disrupt FA homeostasis, but there are no epidemiological data regarding associations of PFCs and FA concentrations.

**Objectives:**

We estimated associations between perfluorooctane sulfonate (PFOS)/perfluorooctanoate (PFOA) concentrations and maternal levels of FAs and triglyceride (TG) and birth size of the offspring.

**Methods:**

We analyzed 306 mother–child pairs in this birth cohort between 2002 and 2005 in Japan. The prenatal PFOS and PFOA levels were measured in maternal serum samples by liquid chromatography–tandem mass spectrometry. Maternal blood levels of nine FAs and TG were measured by gas chromatography–mass spectrometry and TG E-Test Wako kits, respectively. Information on infants’ birth size was obtained from participant medical records.

**Results:**

The median PFOS and PFOA levels were 5.6 and 1.4 ng/mL, respectively. In the fully adjusted model, including maternal age, parity, annual household income, blood sampling period, alcohol consumption, and smoking during pregnancy, PFOS but not PFOA had a negative association with the levels of palmitic, palmitoleic, oleic, linoleic, α-linolenic, and arachidonic acids (*p* < 0.005) and TG (*p-*value = 0.016). Female infants weighed 186.6 g less with mothers whose PFOS levels were in the fourth quartile compared with the first quartile (95% CI: –363.4, –9.8). We observed no significant association between maternal levels of PFOS and birth weight of male infants.

**Conclusions:**

Our data suggest an inverse association between PFOS exposure and polyunsaturated FA levels in pregnant women. We also found a negative association between maternal PFOS levels and female birth weight.

**Citation:**

Kishi R, Nakajima T, Goudarzi H, Kobayashi S, Sasaki S, Okada E, Miyashita C, Itoh S, Araki A, Ikeno T, Iwasaki Y, Nakazawa H. 2015. The association of prenatal exposure to perfluorinated chemicals with maternal essential and long-chain polyunsaturated fatty acids during pregnancy and the birth weight of their offspring: the Hokkaido Study. Environ Health Perspect 123:1038–1045; http://dx.doi.org/10.1289/ehp.1408834

## Introduction

Perfluorinated chemicals (PFCs) are ubiquitous and stable chemicals widely detected in humans and environment. Contamination of drinking water, house dust, foods, and fish products are the possible major exposure pathways of humans to PFCs (Lau et al. 2007). The most widely studied and detected PFCs are perfluorooctane sulfonate (PFOS) and perfluorooctanoate (PFOA). In 2009, PFOS was added to Annex B of the Stockholm Convention on Persistent Organic Pollutants (POPs) (United Nations Environment Programme 2007). PFOS and PFOA are being voluntarily phased out by several industries, and are being substituted by longer carbon-chain PFCs. Recently, we reported that plasma levels of PFOS and PFOA were generally decreasing in plasma of pregnant Japanese women; however, we observed an increased trend for PFCs with longer carbon chains (Okada et al. 2013). Additionally, PFOS and PFOA are still present in older products, and they are slowly eliminated from the human body, with mean half-lives of 5.4 and 3.8 years for PFOS and PFOA, respectively (Olsen et al. 2007).

Both PFOS and PFOA have been shown to have developmental and reproductive toxicity in animal studies, including early pregnancy loss, reduced fetal weight, and postnatal mortality (Abbott et al. 2007; Luebker et al. 2005). A strong correlation of these compounds has been demonstrated between maternal and cord blood samples in humans, indicating that neonates are exposed to PFCs via the placental passage (Inoue et al. 2004; Monroy et al. 2008). Some epidemiological studies have also reported an association between PFC exposure and poor birth outcomes including decreased birth size (Apelberg et al. 2007; Fei et al. 2007; Washino et al. 2009). In a prospective study, prenatal PFOA exposure was positively associated with the prevalence of overweight female offspring at 20 years of age (Halldorsson et al. 2012). However, Barry et al. (2014) reported no association between early-life PFOA exposure and overweight and obesity risk in adults 20–40 years of age.

Recent research has shown that PFCs perturb metabolic end points, including lipid metabolism, glucose homeostasis, and thyroid hormone balance, in animals (Seacat et al. 2003; Thibodeaux et al. 2003). Such effects might explain associations between PFCs and birth outcomes. Most epidemiological studies regarding the association between PFCs and lipids [triglyceride (TG) and cholesterol] have been conducted in nonpregnant participants. Although previous reports suggest a positive association between PFCs and cholesterol levels (Frisbee et al. 2010; Winquist and Steenland 2014), the reports regarding the association of PFCs and TG levels are inconsistent. In a targeted group of Inuit adults 18–74 years of age, Château-Degat et al. (2010) reported a significant negative association between high PFOS exposure levels and TG only in women. However, some groups reported no association between exposure to PFCs and TG levels (Fisher et al. 2013; Sakr et al. 2007) or even a positive association between exposure to PFCs and TG levels in nonpregnant women (Steenland et al. 2009). Therefore, the relevance of these findings is uncertain.

Fetal growth is dependent on maternal metabolic resources, and this is exemplified by the correlation between maternal and fetal TG and fatty acids (FAs) levels (Kitajima et al. 2001; Schaefer-Graf et al. 2011). Major physiologic changes in lipid metabolism take place during normal pregnancies. There is an increase in body fat depots during early pregnancy, whereas lipolysis of fat depots occurs in late pregnancy, resulting in hyperlipidemia. Maternal hyperlipidemia during late pregnancy facilitates the availability of lipid substrates to the fetus. Fetuses need essential fatty acids (EFAs) and long-chain polyunsaturated fatty acids (LCPUFAs) for growth and development, especially for nervous system development (Alvarez et al. 1996). EFAs include linoleic acid and α-linolenic acid, which are precursors of omega 6 and omega 3 LCPUFAs, respectively. These substances must be obtained from the maternal diet, and the maternal blood concentrations of EFAs determine the corresponding concentrations in the cord blood (Herrera and Ortega-Senovilla 2010).

PFCs resemble FAs structurally and they may disrupt the homeostasis of FAs (Hu et al. 2005). Physiologic hyperlipidemia in late pregnancy is essential for fetal growth. However, to our knowledge, the influence of PFCs on FA homeostasis in pregnant women has not been investigated. In the present analyses, we explored the relationship between PFOS/PFOA exposure and the levels of four families of FAs (saturated, monounsaturated, omega 3, and omega 6 polyunsaturated FAs) including nine FAs and TGs in the maternal blood samples and birth size of their offspring from a birth cohort study.

## Methods

*Study population*. This study was a part of the Hokkaido Study on Environment and Children’s Health conducted between July 2002 and October 2005, and the details have been previously described (Kishi et al. 2011, 2013). Of 1,796 potentially eligible women, the following subjects were excluded: women who decided to participate in the Japanese cord blood bank (22% of those approached), and women who decided to deliver at another hospital (3% of those approached). Of the remaining eligible subjects, 514 women (28.6% of those approached) agreed to participate in this study (Konishi et al. 2009). These pregnant women at 23–35 weeks of gestation registered during a routine gynecologic checkup and delivered at the Toho Hospital in Sapporo, Hokkaido, Japan. Ten registered women were excluded due to miscarriage and stillbirth (*n* = 2), relocation (*n* = 1), or voluntary withdrawal (*n* = 7) from the study before follow-up.

*Questionnaires and medical records.* A self-administered questionnaire survey was completed after the second trimester (Washino et al. 2009) containing information related to smoking, household income and educational levels, and alcohol and caffeine intake during pregnancy. Medical information including maternal age, maternal body mass index (BMI) before pregnancy, parity, gestational age, pregnancy complications, type of delivery, infant sex, and birth size (weight, length, chest, and head circumferences) were obtained from participant medical records. This study was conducted with the written informed consent of all participants, and the study protocol was approved by the institutional ethical board for epidemiological studies at the Graduate School of Medicine and Center for Environmental and Health Sciences, Hokkaido University.

*Blood sampling and exposure assessments.* A 40-mL blood sample was taken from a peripheral vein after the second trimester of pregnancy and was used to measure maternal serum levels of PFOS, PFOA, TG, and FAs. All samples were stored at –80°C until analysis. Detailed methods for the measurement of PFOS and PFOA have been described in our previous report (Nakata et al. 2009). In brief, serum samples (0.1 mL) were mixed with 0.2 mL internal standard (^13^C_4_-PFOS-Na^+^ and ^13^C_2_-PFOA) solution containing acetonitrile, centrifuged at 1,450 × *g* for 10 min, and the supernatant was transferred to a polypropylene tube. An aliquot of the filtered sample solution was subjected to column-switching liquid chromatography–tandem mass spectrometry. The PFOS values of all samples were detected, and for samples with PFOA levels below the detection limit (0.50 ng/mL) (*n* = 17, 5.5% of participants), we used a value of half the detection limit (0.25 ng/mL).

*The TG and FA concentrations in maternal blood.* The FA levels in nonfasting maternal blood specimens were determined by gas chromatography–mass spectrometry (GC-MS) as described previously in detail (Nakashima et al. 2013). Briefly, the FA levels in maternal blood were measured as follows: Lipid extracted from 25 μL of blood according to the method of Folch et al. (1957) was mixed with 1.2 mL methanol, 75 μL acetyl chloride, and 75 μL 10 μg/100 μL tricosanoic acid ethyl ester/methanol (internal standard). After adding *n*-hexane (500 μL) and centrifugation, the upper organic layer was collected and moved into another vial. The *n*-hexane extraction was repeated once more, and then the concentration of FA methyl ester contained in the *n*-hexane layer was measured by GC-MS. Finally, the nine FA species targeted for measurement, including the palmitic and stearic acids of saturated FAs, the palmitoleic and oleic acids of monounsaturated FAs, linoleic acid (LA) and arachidonic acid (AA) of the omega 6 FAs, and the α-linolenic acid (ALA), eicosapentaenoic acid (EPA), and docosahexaenoic acid (DHA) of omega 3 FAs. The detection limits were 2.4 μL/mL for palmitic acid, 1.3 μg/mL for stearic acid, 0.069 μg/mL for palmitoleic acid, 3.6 μg/mL for oleic acid, and 2.0 μg/mL for the others. The detection rates for all FAs were > 99.0% (except for EPA, with a detection rate of 97.8%). Nonfasting blood TG levels were measured using TG E-Test Wako kits (Wako, Osaka, Japan) after lipid extraction according to the methods described by Folch et al. (1957).

*Data analysis.* For the analysis of associations between maternal PFOS and PFOA levels and birth size, the following subjects were excluded: women with pregnancy-induced hypertension (*n* = 11), women with diabetes mellitus (*n* = 1), mother–infant pairs with fetal heart failure (*n* = 1), and twins (*n* = 7). After the exclusion of these subjects, 428 mother–infant pairs had available PFOS and PFOA concentrations. We excluded subjects whose blood samples were obtained after delivery (*n* = 105). Additionally, TG and/or FA levels were not available for 17 subjects. The available sample size for statistical analysis after considering the exclusion criteria was 306 subjects. Because of the skewed distributions, we treated the levels of PFOS, PFOA, and lipids as a continuous variable on a log_10_ scale.

We analyzed correlations between PFOS and PFOA concentrations and the characteristics of the mothers and infants using the Spearman correlation test, the Mann–Whitney *U*-test, and the Kruskal–Wallis test. The same statistical analyses were performed to find correlations between the maternal blood TG and FA levels and the characteristics of the mothers and infants. Additionally, we performed multiple regression analyses to determine the relationship between the maternal PFOS and PFOA levels and the lipid levels, and potential confounders selected according to the current results in this paper influencing exposure levels (maternal age, parity, smoking during pregnancy), lipid levels (alcohol intake during pregnancy), or both (blood sampling period). In addition, we included annual household income as an indicator of socioeconomic status. Therefore, the fully adjusted models included maternal age (years), smoking status and alcohol intake during pregnancy (yes/no), annual household income (categorical), parity (0/≥ 1), and the blood sampling period during pregnancy (categorical or continuous). The blood sampling period during pregnancy was categorized in model 1 as follows: 23–31 weeks of gestation (*n* = 137), 32–34 weeks of gestation (*n* = 82), and 35–41 weeks of gestation (*n* = 87) (Konishi et al. 2009). Due to the importance of the blood sampling time with respect to the PFC and TG/FA levels, we included the blood sampling period as a continuous variable (by week of pregnancy) for tighter adjustment of model 2. Additionally, the daily inshore and deep-sea fish intake were included as potential covariates for the levels of EPA, DHA, and omega 3 FA levels (Grandjean et al. 2001). After log_10_-transformation of PFOS and lipid levels (TG and fatty acids), we divided PFOS levels into four quartiles. Then, least square means (LSMs) and 95% confidence intervals (CIs) were calculated, and the LSMs and CIs were back transformed from log_10_ to normal values. For calculation of *p* for trend, we used linear contrast coefficients –3, –1, +1, +3 assigned to quartiles 1, 2, 3, and 4, respectively. The same approach was applied to find the association between the PFC levels and infant birth size, and confounders as reported by our group previously (Washino et al. 2009) were maternal age, annual household income, alcohol intake and smoking status during pregnancy, maternal BMI, parity, the blood sampling period (categorical), and gestational age (weeks). In addition, the least square means of birth weight for each quartile were compared using the Hsu–Dunnett method to accommodate for multiple comparisons. To examine the association with birth size, neonates were stratified by sex. We performed all of the statistical analyses using JMP pro 10 (SAS Institute Inc., Cary, NC, USA). The results were considered statistically significant at *p* < 0.05.

## Results

Table 1 shows the maternal serum PFOS and PFOA concentrations in relation to the characteristics of the mothers and infants. The median (25–75 percentile) values of PFOS and PFOA were 5.6 ng/mL (4.0–7.5 ng/mL) and 1.4 ng/mL (0.9–2.0 ng/mL), respectively. The Spearman correlation coefficients detected a modest level of correlation between the PFOS and PFOA concentrations (ρ = 0.223, *p*-value < 0.001). We found statistically significant differences in the mean PFOS concentrations by parity, smoking during pregnancy, and the blood sampling period (*p* ≤ 0.001). Additionally, we observed significant differences in the mean PFOA concentrations by parity, caffeine intake during pregnancy, and infant sex.

**Table 1:** Maternal blood PFOS and PFOA levels (ng/mL) in relation to the characteristics of the subjects participating in the Hokkaido Study on Environment and Children’s Health, Sapporo, Japan, 2002–2005 (*n *= 306).

Characteristic	*n* (%)	PFOS [mean ± SD, median(25th–75th percentile),or correlation^*a*^ (*p*-value)]	*p*-Value^*b*^	PFOA [mean ± SD, median(25th–75th percentile),or correlation^*a*^ (*p*-value)]	*p*-Value^*b*^
Mean (± SD)	306 (100.0)	6.02 ± 2.67		1.52 ± 0.89
Median (25th–75th percentile)	306 (100.0)	5.60 (4.0–7.5)		1.40 (0.9–2.0)
Maternal characteristics
Age (years)
< 28	87 (28.4)	6.72 ± 0.28	0.020	1.68 ± 0.09	0.140
28–33	151 (49.4)	5.77 ± 0.21		1.43 ± 0.07
> 34	68 (22.2)	5.69 ± 0.32		1.49 ± 0.10
Prepregnancy BMI (kg/m^2^)
< 18.5	44 (14.4)	6.18 ± 0.40	0.519	1.55 ± 0.13	0.955
18.5–25	236 (77.1)	6.05 ± 0.17		1.51 ± 0.05
> 25	26 (8.5)	5.56 ± 0.52		1.52 ± 0.17
Parity^*c*^
0	161 (52.7)	6.54 ± 0.20	0.001	1.80 ± 0.06	< 0.001
≥ 1	144 (47.2)	5.46 ± 0.21		1.20 ± 0.07
Educational level (years)
≤ 12	138 (45.0)	5.78 ± 0.22	0.195	1.50 ± 0.07	0.330
≥ 13	168 (54.9)	6.22 ± 0.20		1.53 ± 0.06
Annual household income (million yen)^*c*^
< 5	210 (69.0)	5.96 ± 0.18	0.567	1.54 ± 0.06	0.934
> 5	94 (30.9)	6.13 ± 0.27		1.47 ± 0.09
Smoking during pregnancy
Yes	130 (42.4)	5.41 ± 0.23	< 0.001	1.45 ± 0.07	0.353
No	176 (57.5)	6.48 ± 0.19		1.56 ± 0.06
Alcohol consumed during pregnancy
Yes	97 (31.6)	5.95 ± 0.27	0.868	1.45 ± 0.09	0.560
No	209 (68.3)	6.06 ± 0.18		1.55 ± 0.06
Caffeine intake during pregnancy (mg/day)	144.3 ± 123	ρ = –0.058	0.310	ρ = –0.125	0.028
Fish intake during pregnancy
Inshore fish
≤ 1–2 times/month	166 (54.2)	6.00 ± 0.20	0.750	1.55 ± 0.06	0.996
≥ 1–2 times/week	140 (45.7)	6.05 ± 0.22		1.48 ± 0.07
Deep-sea fish
≤ 1–2 times/month	148 (48.3)	5.91 ± 0.22	0.223	1.52 ± 0.07	0.958
≥ 1–2 times/week	158 (51.6)	6.14 ± 0.21		1.51 ± 0.07
Blood sampling period
23–31 weeks during pregnancy	137 (44.7)	6.35 ± 0.22	< 0.001	1.59 ± 0.07	0.079
32–34 weeks during pregnancy	82 (26.7)	6.51 ± 0.28		1.49 ± 0.09
35–41 weeks during pregnancy	87 (28.4)	5.05 ± 0.27		1.43 ± 0.09
Type of delivery
Vaginal	255 (83.3)	6.14 ± 0.16	0.109	1.52 ± 0.05	0.915
Cesarean section	51 (16.6)	5.45 ± 0.37		1.47 ± 0.12
Gestational age (days)	276.7 ± 9.4	ρ = 0.057	0.314	ρ = 0.051	0.371
Infant characteristics
Sex
Male	141 (46.0)	6.18 ± 0.22	0.409	1.59 ± 0.07	0.040
Female	165 (53.9)	5.89 ± 0.20		1.45 ± 0.06
Birth weight (g)
Total	3076.9 ± 377.4	ρ = –0.067	0.238	ρ = –0.117	0.039
Male	3093.6 ± 374.2	ρ = 0.075	0.372	ρ = –0.076	0.367
Female	3062.5 ± 380.6	ρ = –0.185	0.017	ρ = –0.148	0.056
^***a***^Spearman’s correlation (ρ) and *p*-value for continuous variables. ^***b***^*p*-Values for Mann–Whitney *U*-test or Kruskal–Wallis test. ^***c***^Missing data: parity (*n *= 1), annual household income (*n *= 2).					

The maternal blood levels of TG and nine FA and their relation to the maternal and infant characteristics are shown in Table 2. The EPA levels increased with the higher frequency of inshore fish intake (*p <* 0.001). Maternal TG, palmitic acid, palmitoleic acid, and oleic acid increased significantly with increased gestation, but, inversely, DHA decreased with increased gestation. Maternal age, caffeine intake during pregnancy, and maternal education levels did not show significant associations with maternal lipids (data not shown). Table 3 presents the results of the univariate and multivariate regression analyses for the maternal blood TG and FAs on the log_10_-transformed PFOS and PFOA concentrations. In the crude model, we found significant negative associations of PFOS exposure with TG, palmitic acid, and the palmitoleic and the oleic acids of monounsaturated FAs, LA and AA of the omega 6 family, and ALA of the omega 3 family in maternal blood samples (*p* < 0.01). We considered two models for adjustment; in both models, maternal age, smoking and alcohol intake during pregnancy, annual household income, parity, and the blood sampling period (categorical in model 1; continuous in model 2) were used as confounding factors. In model 2, due to the importance of the blood sampling period for the PFC and lipid levels, blood sampling was included as a continuous variable (by week of pregnancy) for tighter adjustment. After adjusting models 1 and 2, for which the outcome and exposure levels were both log_10_ transformed, significant associations remained for TG and all six mentioned FAs. In model 2, PFOS was negatively associated with the TG/FAs levels as follows: TG (β = –0.130; 95% CI: –0.253, –0.011), palmitic acid (β = –0.175; 95% CI: –0.240, –0.044), palmitoleic acid (β = –0.168; 95% CI: –0.338, –0.058), oleic acid (β = –0.149; 95% CI: –0.242, –0.026), linoleic acid (β = –0.278; 95% CI: –0.745, –0.294), α-linolenic acid (β = –0.227; 95% CI: –0.739, –0.220), and arachidonic acid (β = –0.184; 95% CI: –0.555, –0.111). Additionally, the prenatal PFOS levels were significantly negatively associated with EFAs (β = –0.278; 95% CI: –0.745, –0.294) and omega 6 FAs (β = –0.272; 95% CI: –0.722, –0.277). No significant associations between PFOS and stearic acid and EPA were detected in any model. We did not observe any significant association between PFOA and lipid levels, except a positive association of prenatal PFOA levels with palmitic acid after full adjustment (β = 0.136; 95% CI: 0.009, 0.152).

**Table 2:** Maternal blood TG and FA levels in relation to the characteristics of the mother–infant pairs (*n *= 306).

Characteristic	*n*	TG(mg/dL)	Palmitic acid (μg/mL)	Palmitoleic acid (μg/mL)	Stearic acid (μg/mL)	Oleic acid (μg/mL)	Linoleic acid(μg/mL)	α‑Linolenic acid (μg/mL)	Arachidonic acid (μg/mL)	EPA(μg/mL)	DHA (μg/mL)
All samples	306	90.8 ± 46.1	2064.8 ± 858.8	126 ± 78.5	573.1 ± 206.3	1214.5 ± 564.5	721.2 ± 419.7	10.7 ± 8.3	71.2 ± 42.4	10.2 ± 8.4	30.2 ± 21.6
Maternal characteristics
Parity^*a*^^,^^*b*^
0	161	88.5 ± 3.6	2063.0 ± 67.8	123.4 ± 6.2	582.0 ± 16.2	1207.4 ± 44.6	711.9 ± 33.1	10.5 ± 0.6	71.0 ± 3.3	10.1 ± 0.6	29.0 ± 1.7
≥ 1	144	92.7 ± 3.8	2070.1 ± 71.7	129.2 ± 6.5	564.5 ± 17.2	1223.5 ± 47.1	729.7 ± 35.0	10.9 ± 0.6	71.4 ± 3.5	10.2 ± 0.7	31.4 ± 1.8
Smoking during pregnancy^*a*^
Yes	130	95.7 ± 4.0	2121.9 ± 75.3	133.3 ± 6.8	563.5 ± 18.1	1268.9 ± 49.4	740.5 ± 36.8	10.9 ± 0.7	69.0 ± 3.7	9.1 ± 0.7	27.4 ± 1.8
No	176	87.1 ± 3.4	2022.6 ± 64.7	120.6 ± 5.9	580.2 ± 15.5	1174.4 ± 42.4	707.0 ± 31.6	10.6 ± 0.6	72.8 ± 3.1	11.0 ± 0.6	32.2 ± 1.6
Alcohol intake during pregnancy^*a*^
Yes	97	83.8 ± 4.6	2020.1 ± 87.2	118.4 ± 7.9	573.7 ± 20.9	1200.5 ± 57.4	745.0 ± 42.6	11.4 ± 0.8	75.2 ± 4.3	10.2 ± 0.8	32.2 ± 2.2
No	209	94.0 ± 3.1*	2085.6 ± 59.4	129.5 ± 5.4	572.8 ± 14.2	1221.0 ± 39.1	710.2 ± 29.0	10.4 ± 0.5	69.4 ± 2.9	10.2 ± 0.5	29.3 ± 1.5
Fish intake during pregnancy^*a*^
Inshore fish
≤ 1–2 times/month	166	91.0 ± 3.5	2054.1 ± 66.7	127.6 ± 6.1	566.3 ± 16.0	1217.7 ± 43.8	708.5 ± 32.6	10.2 ± 0.64	71.8 ± 3.2	8.8 ± 0.64	28.6 ± 1.6
≥ 1–2 times/week	140	90.5 ± 3.9	2077.5 ± 72.6	124.0 ± 6.6	581.1 ± 17.4	1210.8 ± 47.7	736.4 ± 35.5	11.3 ± 0.70	70.5 ± 3.5	11.8 ± 0.7**	32.1 ± 1.8
Deep-sea fish
≤ 1–2 times/month	148	91.6 ± 3.7	2114.6 ± 70.5	132.9 ± 6.4	586.9 ± 16.9	1248.7 ± 46.4	723.7 ± 34.5	10.7 ± 0.6	72.0 ± 3.4	9.8 ± 0.6	28.8 ± 1.7
≥ 1–2 times/week	158	90.0 ± 3.6	2018.1 ± 68.3	119.5 ± 6.2	560.2 ± 16.4	1182.5 ± 44.9	718.9 ± 33.4	10.7 ± 0.6	70.5 ± 3.3	10.5 ± 0.6	31.5 ± 1.7
Blood sampling period (categorical)^*c*^
23–31 weeks during pregnancy	137	81.2 ± 3.8	1928.7 ± 72.7	110.7 ± 6.6	562.7 ± 17.4	1095.8 ± 47.3	686.3 ± 35.8	10.3 ± 0.7	70.9 ± 3.5	10.5 ± 0.7	32.3 ± 1.8
32–34 weeks during pregnancy	82	93.5 ± 5.0	2107.2 ± 94.0	129.5 ± 8.5	624.2 ± 22.5	1252.1 ± 61.2	726.6 ± 46.3	10.9 ± 0.9	80.1 ± 4.6	10.1 ± 0.9	32.0 ± 2.3
35–41 weeks during pregnancy	87	103.2 ± 4.8**	2239.2 ± 91.2*	146.7 ± 8.2*	541.2 ± 21.9**	1366.2 ± 59.4**	771.3 ± 44.9	11.1 ± 0.8	63.4 ± 4.5	9.7 ± 0.9	25.2 ± 2.3*
Blood sampling period (week of pregnancy)^*d*^	306	ρ = 0.198**	ρ = 0.176**	ρ = 0.208**	ρ = 0.055	ρ = 0.223**	ρ = 0.086	ρ = 0.035	ρ = –0.042	ρ = –0.009	ρ = –0.122*
Infant characteristics
Birth weight^*d*^	306	ρ = –0.007	ρ = –0.055	ρ = –0.076	ρ = –0.074	ρ = –0.011	ρ = 0.021	ρ = –0.022	ρ = 0.010	ρ = 0.052	ρ = 0.031
Abbreviations: DHA, docosahexaenoic acid; EPA, eicosapentaenoic acid; TG, triglyceride. Data are means ± SD or correlations unless otherwise indicated.*p < 0.05. **p < 0.01. *p*-Values were calculated by ^***a***^Mann–Whitney *U*-test, ^***c***^Kruskal–Wallis test, or ^***d***^Spearman’s correlation (ρ); ^***b***^missing data: parity (*n *= 1);											

**Table 3:** The regression coefficients (95% CIs) between PFOS/PFOA concentrations (ng/mL) and the levels of TG and FAs in the maternal blood (*n *= 306).

Dependent variable	PFOS [β (95% CI)]	*p*-Value	PFOA [β (95% CI)]	*p*-Value
TG
Crude	–0.197 (–0.313, –0.087)	< 0.001	0.008 (–0.078, 0.090)	0.888
Model 1	–0.147 (–0.272, –0.027)	0.016	0.059 (–0.045, 0.134)	0.333
Model 2	–0.130 (–0.253, –0.011)	0.032	0.066 (–0.039, 0.138)	0.273
Palmitic acid
Crude	–0.215 (–0.264, 0.085)	< 0.001	0.084 (–0.016, 0.117)	0.138
Model 1	–0.204 (–0.264, –0.067)	0.001	0.126 (0.002, 0.147)	0.043
Model 2	–0.175 (–0.240, –0.044)	0.004	0.136 (0.009, 0.152)	0.027
Palmitoleic acid
Crude	–0.227 (–0.397, –0.138)	< 0.001	–0.008 (–0.104, 0.090)	0.886
Model 1	–0.195 (–0.371, –0.088)	0.001	0.048 (–0.063, 0.146)	0.436
Model 2	–0.168 (–0.339, –0.058)	0.005	0.059 (–0.052, 0.155)	0.333
Stearic acid
Crude	0.047 (–0.046, 0.112)	0.410	0.068 (–0.022, 0.093)	0.229
Model 1	0.017 (–0.074, –0.098)	0.779	0.055 (–0.034, 0.091)	0.374
Model 2	0.056 (–0.047, 0.126)	0.372	0.058 (–0.033, 0.094)	0.352
Oleic acid
Crude	–0.214 (–0.291, –0.093)	< 0.001	0.051 (–0.040, 0.107)	0.369
Model 1	–0.179 (–0.270, –0.052)	0.003	0.101 (–0.013, 0.147)	0.102
Model 2	–0.149 (–0.242, –0.026)	0.014	0.112 (–0.055, 0.153	0.067
Linoleic acid
Crude	–0.264 (–0.701, –0.292)	< 0.001	0.036 (–0.104, 0.205)	0.524
Model 1	–0.295 (–0.777, –0.326)	< 0.001	0.051 (–0.100, 0.240)	0.420
Model 2	–0.278 (–0.745, –0.294)	< 0.001	0.055 (–0.095, 0.245)	0.385
α-Linolenic acid
Crude	–0.222 (–0.705, –0.237)	< 0.001	0.010 (–0.158, 0.192)	0.849
Model 1	–0.248 (–0.782, –0.263)	< 0.001	0.021 (–0.160, 0.226)	0.735
Model 2	–0.227 (–0.739, –0.220)	< 0.001	0.026 (–0.152, 0.234)	0.675
Arachidonic acid
Crude	–0.146 (–0.467, –0.062)	0.010	–0.017 (–0.173, 0.126)	0.756
Model 1	–0.201 (–0.584, –0.142)	0.001	–0.030 (–0.204, 0.123)	0.628
Model 2	–0.184 (–0.555, –0.111)	0.003	–0.031 (–0.206, 0.123)	0.619
EPA
Crude	0.111 (–0.0004, 0.426)	0.054	0.017 (–0.133, 0.180)	0.764
Model 1^*a*^	0.085 (–0.072, 0.398)	0.175	0.007 (–0.160, 0.179)	0.908
Model 2^*a*^	0.101 (–0.041, 0.426)	0.107	0.011 (–0.154, 0.186)	0.854
DHA
Crude	–0.062 (–0.339, 0.098)	0.278	–0.040 (–0.218, 0.102)	0.477
Model 1^*a*^	–0.120 (–0.472, 0.005)	0.052	–0.055 (–0.253, 0.096)	0.377
Model 2^*a*^	–0.103 (–0.440, 0.040)	0.102	–0.055 (–0.255, 0.097)	0.377
Essential fatty acids
Crude	–0.264 (–0.700, –0.291)	< 0.001	0.036 (–0.104, 0.205)	0.521
Model 1	–0.295 (–0.776, –0.326)	< 0.001	0.051 (–0.100, 0.240)	0.419
Model 2	–0.278 (–0.745, –0.294)	< 0.001	0.055 (–0.095, 0.246)	0.384
Omega 6
Crude	–0.256 (–0.676, –0.272)	< 0.001	0.032 (–0.108, 0.196)	0.571
Model 1	–0.289 (–0.753, –0.309)	< 0.001	0.045 (–0.106, 0.228)	0.474
Model 2	–0.272 (–0.722, –0.277)	< 0.001	0.049 (–0.102, 0.234)	0.440
Omega 3
Crude	–0.041 (–0.225, 0.104)	0.473	–0.034 (–0.158, 0.083)	0.543
Model 1^*a*^	–0.090 (–0.314, 0.047)	0.149	–0.047 (–0.182, 0.081)	0.452
Model 2^*a*^	–0.069 (–0.283, 0.079)	0.268	–0.044 (–0.180, 0.085)	0.479
Abbreviations: DHA, docosahexaenoic acid; EPA, eicosapentaenoic acid; TG, triglyceride. Data are log_10_-transformed exposure and outcomes. Units: TG (md/dL); FAs (μg/mL). Model 1 was adjusted for maternal age, smoking and alcohol intake during pregnancy, annual household income, parity, and blood sampling period (categorical). Model 2 was adjusted for maternal age, smoking and alcohol intake during pregnancy, annual household income, parity, and blood sampling period (by week of pregnancy).^***a***^For EPA, DHA, and omega 3 FAs, adjusted model 1 and 2 also included fish intake.				

**Figure 1:**
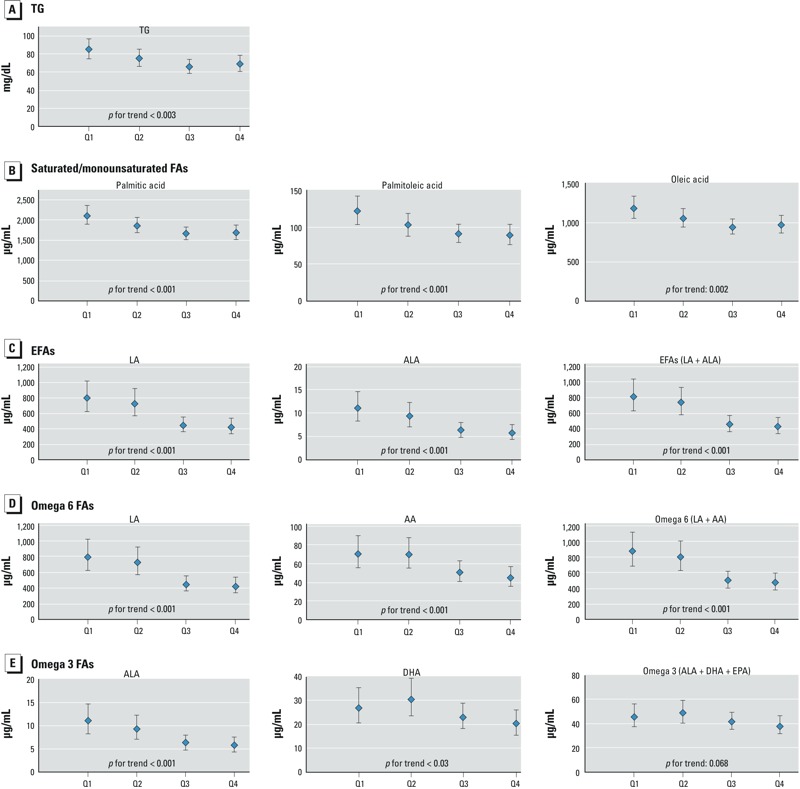
The dose–response relationship between the quartiles (Q) of PFOS and reduced levels of TG (*A*) and FAs (*B*–*E*) in maternal serum samples, Sapporo, Japan, 2002–2005 (*n *= 306). The LSMs were adjusted for maternal age, smoking and alcohol intake during pregnancy, annual household income, parity, and the blood sampling period (categorical). For EPA, DHA, and omega 3 FAs, the adjusted model also included fish intake. The LSMs were back transformed from log_10_ to normal values, and the error bars depict 95% CIs.

We also examined the association between the plasma PFOS quartiles, TG and FAs. The PFOS concentrations were divided into quartiles: 1.5–4.0, 4.0–5.6, 5.6–7.5, and 7.5–16.2 ng/mL. In Figure 1 (see also Supplemental Material, Table S1), the quartile analysis after full adjustment showed decreasing trends for lipids in the fourth quartile of PFOS compared with the first quartile, with significant linear trend: TG (–16.1 mg/dL, *p* for trend < 0.003), palmitic acid (–422.1 μg/mL, *p* for trend < 0.001), palmitoleic acid (–32.4 μg/mL, *p* for trend < 0.001), oleic acid (–217.5 μg/mL, *p* for trend = 0.002), LA (–373.6 μg/mL, *p* for trend < 0.001), ALA (–5.2 μg/mL, *p* for trend < 0.001), AA (–24.9 μg/mL, *p* for trend < 0.001) and DHA (–6.4 μg/mL, *p* for trend < 0.03). In addition, increasing PFOS quartiles were negatively associated with EFA (–379.5 μg/mL, *p* for trend < 0.001) and omega 6 FAs (–399.7 μg/mL, *p* for trend < 0.001). However, a nonsignificant negative association was observed between the PFOS levels and omega 3 FAs (–7.3 μg/mL, *p* for trend = 0.068).

Maternal PFOS concentrations were negatively associated with birth weight among female infants but not male infants (Table 4). In the adjusted model, female infants whose mothers were in the highest quartile of PFOS concentration weighed –186.6 g less (95% CI: –363.4, –9.8) than female infants born to mothers with concentrations in the lower quartile. PFOS was not significantly associated with birth length, or with chest or head circumference in either sex (data not shown). Additionally, we did not observe a significant association between prenatal PFOA exposure and any of the birth size outcomes (data not shown).

**Table 4:** The crude and adjusted least square means (LSMs) and regression coefficients (β) for the birth weight (g) of male and female infants by quartiles of PFOS.

PFOS in quartiles (ng/mL)	Crude		Adjusted^*a*^	
	LSM	β (95% CI)	LSM	β (95% CI)
Male infants (*n *= 141)
Quartile 1 (1.60–4.35)	3059.5	Reference	3069.2	Reference
Quartile 2 (4.35–5.80)	3010.6	–48.9 (–267.6, 169.8)	3012.4	–56.7 (–255.9, 142.4)
Quartile 3 (5.80–7.45)	3151.3	91.7 (–123.3, 306.8)	3099.7	30.5 (–169.7, 230.8)
Quartile 4 (7.45–16.20)	3144.1	84.5 (–136.9, 306.0)	3165.1	95.9 (–116.5, 308.4)
*p* for trend	0.182		0.187
Female infants (*n *= 165)
Quartile 1 (1.50–3.80)	3147.0	Reference	3125.4	Reference
Quartile 2 (3.80–5.50)	3034.6	–112.4 (–310.5, 85.6)	3055.2	–70.1 (–242.5, 102.2)
Quartile 3 (5.50–7.65)	3120.1	–26.9 (–217.7, 163.9)	3086.3	–39.1 (–216.1, 137.8)
Quartile 4 (7.65–13.30)	2934.6	–212.3 (–400.7, –24.0)*		–186.6 (–363.4, –9.8)*
*p* for trend	0.030		0.031
^***a***^Adjusted for gestational age, maternal age, prepregnancy BMI, smoking and drinking during pregnancy, parity, annual household income, and blood sampling period (categorical). *p < 0.05 compared with quartile 1 calculated by Hsu–Dunnett method.				

## Discussion

To our knowledge, this study is the first to address the association between PFCs and FAs in pregnant women. In our analysis, we found a significant negative association of relatively low PFOS levels with maternal TG and several FA levels including saturated (palmitic acid), monounsaturated (palmitoleic and oleic acids), omega 6 (LA and AA), and omega 3 (ALA) fatty acids during pregnancy in a Japanese birth cohort. There was a significant decreasing trend for TG, palmitic acid, palmitoleic acid, oleic acid, LA, ALA, AA, and DHA with increasing PFOS levels. Javins et al. (2013) reported a consistent decreasing trend of PFCs throughout all trimesters for pregnancy. Additionally, the levels of maternal TG and FAs increased in late pregnancy, consistent with the supply of the fetal levels (Herrera and Ortega-Senovilla 2010). To reduce the potential for bias due to confounding by sampling time, we included the blood sampling period as a continuous variable (by week of pregnancy) in the fully adjusted model (model 2), which had little influence on estimated associations. Our results provide important evidence regarding the association of low background levels of PFOS with the concentration of essential and long-chain polyunsaturated fatty acids. Furthermore, prenatal PFOS exposure was negatively associated with birth weight in female newborns. Unlike PFOS, the levels of PFOA did not have strong associations with the lipid levels or birth size.

Median concentrations for maternal PFOS and PFOA in the current study were 5.60 and 1.40 ng/mL, respectively, which are lower than the median values of cohorts conducted in the United States (PFOS: 8.2 ng/mL, PFOA: 2.9 ng/mL) (Stein et al. 2012), Canada (PFOS: 16.6 ng/mL, PFOA: 2.1 ng/mL) (Monroy et al. 2008), Denmark (PFOS: 21.5 ng/mL, PFOA: 3.7 ng/mL) (Halldorsson et al. 2012), Norway (PFOS: 13 ng/mL, PFOA: 2.2 ng/mL) (Starling et al. 2014), Korea (PFOS: 9.3 ng/mL, PFOA: 2.6 ng/mL) (Lee et al. 2013), and China (PFOS: 6.7 ng/mL, PFOA: 4 ng/mL) (Jiang et al. 2014) among pregnant women.

To our knowledge, the association between PFCs and human FA levels has not been described prior to this study. Two studies described the association between PFCs and TG during pregnancy. In the Baltimore THREE Study, Apelberg et al. (2007) reported a negative association between cord blood PFOS and PFOA levels (median, 5 ng/mL and 1.6 ng/mL, respectively) and birth weight, the ponderal index, and head circumference in a cross-sectional study. However, they did not find an association between PFCs and cord blood TG and total cholesterol levels. Additionally, the associations between PFOS/PFOA and birth size persisted after adjustment for cord serum TG and cholesterol concentrations. In another cross-sectional study in Norway, no associations between maternal PFOS (median, 13 ng/mL) and PFOA (median, 2.2 ng/mL) and TG levels during pregnancy were found (Starling et al. 2014).

In contrast with our findings, Steenland et al. (2009) reported that serum TGs were positively associated with higher deciles of PFOS and PFOA in a cross-sectional study of adults living in close proximity to a chemical plant, with no statistical evidence of effect modification by sex. However, exposure levels in the previous study were substantially higher (median values of 19.6 and 26.6 ng/mL for PFOS and PFOA, respectively) than the low background levels in our study population (median values of 5.6 ng/mL and 1.4 ng/mL, respectively). In contrast, Château-Degat et al. (2010) reported a significant negative association between PFOS exposure levels (arithmetic mean, 23.1 ng/mL) and TG levels among nonpregnant women in an Inuit population of Nunavik (*n* = 723). Similarly, we observed a negative association between low PFOS exposure and maternal TG levels during pregnancy. In rodent studies, PFOS exposures were reported to reduce TG serum concentrations (Seacat et al. 2002, 2003; Thibodeaux et al. 2003), consistent with our findings.

A mode of action(s) that might explain the correlation between the PFC exposure and lipids concentration is not fully understood. Although there is weak activation of PPAR (peroxisome proliferator–activated receptor) γ, the most likely target of PFCs has been shown to be PPARα (Vanden Heuvel et al. 2006; Takacs and Abbott 2007). Curran et al. 2008 showed in rats that PFOS activated PPARα and its downstream genes involved in lipid metabolism, thus resulting in decreased serum triglyceride. Therefore, further studies are needed to clarify the effects of PPAR family polymorphisms on human lipid profile. Unlike PFOS, our estimates did not support an association between *in utero* exposure to PFOA and the levels of FAs (except a positive association with the levels of palmitic acid). Experimental studies suggested that PFOA is a stronger agonist than PFOS for the transactivation of PPARα and PPARγ in mouse and human cells (Vanden Heuvel et al. 2006). PFOS has the highest inhibitory effect on the binding affinity of the liver fatty acid–binding protein for liver FAs whereas PFOA had the lowest potency among the examined PFCs (Luebker et al. 2002). Therefore, PFOS and PFOA may regulate lipid homeostasis with different potencies and modes of action. The maternal serum concentration of PFOA in this study was low, and the differences in the PFOS and PFOA concentrations could also be an alternative explanation.

In this study, we found a significant negative association between the levels of PFOS and female infant birth weight, but not among male infants. However, prenatal PFOA levels did not show significant association with birth weight. Apelberg et al. (2007) found a negative association between the cord blood levels of PFOS and PFOA and the birth weight of infants. Additionally, Maisonet et al. (2012) reported a negative association between the prenatal PFOS and PFOA concentrations and the birth weight of British girls. It is difficult to compare our results with those of other studies because of the differences in the biological samples used to measurement exposure, and the genetic background of our population; however, we found a negative association between prenatal PFOS levels and birth weight, especially in females, consistent with some previous reports (Apelberg et al. 2007; Maisonet et al. 2012). TG and FAs are sources of energy for fetuses, and FA deficiency during fetal development results in metabolic and energy programming (Innis 2011). Although the extent to which these decreases in maternal blood FAs during the gestational period are clinically important to mothers is not apparent, the consequences of hypolipidemia during pregnancy on the growth, neurodevelopment, and metabolic end points of infants should be examined in the future.

This study has some potential limitations. Our study employed only a single measurement of TG and FA concentrations for each participant. Another potential limitation of this study is that we measured the lipids in nonfasting blood samples, and fasting is a routine process before the measurement of lipid profiles. However, recent data show that lipoproteins and lipids, including TG, change minimally following normal food intake (Langsted et al. 2008). Although this study is part of a birth cohort study, the analysis of maternal FAs and TG during pregnancy is cross-sectional in nature, thereby limiting causal inference. Potential selection bias may have occurred because this cohort was based in one hospital that cared for pregnant women in Sapporo and the surrounding areas. Additionally, the participation rate was low due to the exclusion of eligible women who decided to participate in the Japanese cord blood bank. Additionally, before data analysis, we excluded subjects whose blood samples were obtained after delivery (*n* = 105). The excluded subjects had annual household income levels, maternal education levels, and alcohol consumption similar to those of participants in this study, but the incidence of smoking and multiparity was higher, which may suggest the possibility of selection bias.

Previously, our group reported time trends of 11 types of PFCs between 2003 and 2011 in plasma samples of pregnant women in Hokkaido (Okada et al. 2013). The results indicated that PFOS and PFOA concentrations declined, whereas long-chain PFNA and PFDA levels increased. Thus, focusing on the effects of PFCs with longer carbon chain—which may be more potent than ones with shorter chains on TGs, FAs, and other metabolic end points in pregnant women and their offspring—should be included in future studies.

## Conclusions

This study supports the association between PFOS and lipid levels during pregnancy. We found that relatively low PFOS levels had a significant negative association with TG and saturated (palmitic acid), monounsaturated (palmitoleic and oleic acids), omega 6 (linoleic and arachidonic acids), and omega 3 (α-linolenic acid and DHA) FAs.

## Supplemental Material

(139 KB) PDF

## References

[r1] Abbott BD, Wolf CJ, Schmid JE, Das KP, Zehr RD, Helfant L (2007). Perfluorooctanoic acid induced developmental toxicity in the mouse is dependent on expression of peroxisome proliferator activated receptor-alpha.. Toxicol Sci.

[r2] Alvarez JJ, Montelongo A, Iglesias A, Lasunción MA, Herrera E (1996). Longitudinal study on lipoprotein profile, high density lipoprotein subclass, and postheparin lipases during gestation in women.. J Lipid Res.

[r3] Apelberg BJ Witter FR Herbstman JB Calafat AM Halden RU Needham LL 2007 Cord serum concentrations of perfluorooctane sulfonate (PFOS) and perfluorooctanoate (PFOA) in relation to weight and size at birth. Environ Health Perspect 115 1670 1676; 10.1289/ehp.10334 18008002 PMC2072847

[r4] Barry V, Darrow LA, Klein M, Winquist A, Steenland K (2014). Early life perfluorooctanoic acid (PFOA) exposure and overweight and obesity risk in adulthood in a community with elevated exposure.. Environ Res.

[r5] Château-Degat ML, Pereg D, Dallaire R, Ayotte P, Dery S, Dewailly É (2010). Effects of perfluorooctanesulfonate exposure on plasma lipid levels in the Inuit population of Nunavik (Northern Quebec).. Environ Res.

[r6] Curran I, Hierlihy SL, Liston V, Pantazopoulos P, Nunnikhoven A, Tittlemier S (2008). Altered fatty acid homeostasis and related toxicologic sequelae in rats exposed to dietary potassium perfluorooctanesulfonate (PFOS).. J Toxicol Environ Health A.

[r7] Fei C McLaughlin JK Tarone RE Olsen J 2007 Perfluorinated chemicals and fetal growth: a study within the Danish National Birth Cohort. Environ Health Perspect 115 1677 1682; 10.1289/ehp.10506 18008003 PMC2072850

[r8] Fisher M, Arbuckle TE, Wade M, Haines DA (2013). Do perfluoroalkyl substances affect metabolic function and plasma lipids?—analysis of the 2007–2009, Canadian Health Measures Survey (CHMS) Cycle 1.. Environ Res.

[r9] Folch J, Lees M, Sloane Stanley GH (1957). A simple method for the isolation and purification of total lipides from animal tissues.. J Biol Chem.

[r10] Frisbee SJ, Shankar A, Knox SS, Steenland K, Savitz DA, Fletcher T (2010). Perfluorooctanoic acid, perfluorooctanesulfonate, and serum lipids in children and adolescents: results from the C8 Health Project.. Arch Pediatr Adolesc Med.

[r11] Grandjean P, Bjerve KS, Weihe P, Steuerwald U (2001). Birthweight in a fishing community: significance of essential fatty acids and marine food contaminants.. Int J Epidemiol.

[r12] Halldorsson TI Rytter D Haug LS Bech BH Danielsen I Becher G 2012 Prenatal exposure to perfluorooctanoate and risk of overweight at 20 years of age: a prospective cohort study. Environ Health Perspect 120 668 673; 10.1289/ehp.1104034 22306490 PMC3346773

[r13] Herrera E, Ortega-Senovilla H (2010). Disturbances in lipid metabolism in diabetic pregnancy—are these the cause of the problem?. Best Pract Res Clin Endocrinol Metab.

[r14] Hu W, Jones PD, Celius T, Giesy JP (2005). Identification of genes responsive to PFOS using gene expression profiling.. Environ Toxicol Pharmacol.

[r15] Innis SM (2011). Metabolic programming of long-term outcomes due to fatty acid nutrition in early life.. Matern Child Nutr.

[r16] Inoue K Okada F Ito R Kato S Sasaki S Nakajima S 2004 Perfluorooctane sulfonate (PFOS) and related perfluorinated compounds in human maternal and cord blood samples: assessment of PFOS exposure in a susceptible population during pregnancy. Environ Health Perspect 112 1204 1207; 10.1289/ehp.6864 15289168 PMC1247483

[r17] Javins B, Hobbs G, Ducatman AM, Pilkerton C, Tacker D, Knox SS (2013). Circulating maternal perfluoroalkyl substances during pregnancy in the C8 Health Study.. Environ Sci Technol.

[r18] Jiang W, Zhang Y, Zhu L, Deng J (2014). Serum levels of perfluoroalkyl acids (PFAAs) with isomer analysis and their associations with medical parameters in Chinese pregnant women.. Environ Int.

[r19] Kishi R, Kobayashi S, Ikeno T, Araki A, Miyashita C, Itoh S (2013). Ten years of progress in the Hokkaido birth cohort study on environment and children’s health: cohort profile—updated 2013.. Environ Health Prev Med.

[r20] Kishi R, Sasaki S, Yoshioka E, Yuasa M, Sata F, Saijo Y (2011). Cohort profile: the Hokkaido study on environment and children’s health in Japan.. Int J Epidemiol.

[r21] Kitajima M, Oka S, Yasuhi I, Fukuda M, Rii Y, Ishimaru T (2001). Maternal serum triglyceride at 24–32 weeks’ gestation and newborn weight in nondiabetic women with positive diabetic screens.. Obstet Gynecol.

[r22] Konishi K, Sasaki S, Kato S, Ban S, Washino N, Kajiwara J (2009). Prenatal exposure to PCDDs/PCDFs and dioxin-like PCBs in relation to birth weight.. Environ Res.

[r23] Langsted A, Freiberg JJ, Nordestgaard BG (2008). Fasting and nonfasting lipid levels: influence of normal food intake on lipids, lipoproteins, apolipoproteins, and cardiovascular risk prediction.. Circulation.

[r24] Lau C, Anitole K, Hodes C, Lai D, Pfahles-Hutchens A, Seed J (2007). Perfluoroalkyl acids: a review of monitoring and toxicological findings.. Toxicol Sci.

[r25] Lee YJ, Kim MK, Bae J, Yang JH (2013). Concentrations of perfluoroalkyl compounds in maternal and umbilical cord sera and birth outcomes in Korea.. Chemosphere.

[r26] Luebker DJ, Hansen KJ, Bass NM, Butenhoff JL, Seacat AM (2002). Interactions of fluorochemicals with rat liver fatty acid-binding protein.. Toxicology.

[r27] Luebker DJ, York RG, Hansen KJ, Moore JA, Butenhoff JL (2005). Neonatal mortality from in utero exposure to perfluorooctanesulfonate (PFOS) in Sprague–Dawley rats: dose–response, and biochemical and pharamacokinetic parameters.. Toxicology.

[r28] Maisonet M Terrell ML McGeehin MA Christensen KY Holmes A Calafat AM 2012 Maternal concentrations of polyfluoroalkyl compounds during pregnancy and fetal and postnatal growth in British girls. Environ Health Perspect 120 1432 1437; 10.1289/ehp.1003096 22935244 PMC3491920

[r29] Monroy R, Morrison K, Teo K, Atkinson S, Kubwabo C, Stewart B (2008). Serum levels of perfluoroalkyl compounds in human maternal and umbilical cord blood samples.. Environ Res.

[r30] Nakashima R, Hayashi Y, Md K, Jia X, Wang D, Naito H (2013). Exposure to DEHP decreased four fatty acid levels in plasma of prepartum mice.. Toxicology.

[r31] Nakata A, Saito K, Iwasaki Y, Ito R, Kishi R, Nakazawa H (2009). Determination of perfluorinated compounds in human milk and evaluation of their transition from maternal plasma [in Japanese].. Bunseki Kagaku.

[r32] Okada E, Kashino I, Matsuura H, Sasaki S, Miyashita C, Yamamoto J (2013). Temporal trends of perfluoroalkyl acids in plasma samples of pregnant women in Hokkaido, Japan, 2003–2011.. Environ Int.

[r33] Olsen GW Burris JM Ehresman DJ Froehlich JW Seacat AM Butenhoff JL 2007 Half-life of serum elimination of perfluorooctanesulfonate, perfluorohexanesulfonate, and perfluorooctanoate in retired fluorochemical production workers. Environ Health Perspect 115 1298 1305; 10.1289/ehp.10009 17805419 PMC1964923

[r34] Sakr CJ, Kreckmann KH, Green JW, Gillies PJ, Reynolds JL, Leonard RC (2007). Cross-sectional study of lipids and liver enzymes related to a serum biomarker of exposure (ammonium perfluorooctanoate or APFO) as part of a general health survey in a cohort of occupationally exposed workers.. J Occup Environ Med.

[r35] Schaefer-Graf UM, Meitzner K, Ortega-Senovilla H, Graf K, Vetter K, Abou-Dakn M (2011). Differences in the implications of maternal lipids on fetal metabolism and growth between gestational diabetes mellitus and control pregnancies.. Diabet Med.

[r36] Seacat AM, Thomford PJ, Hansen KJ, Clemen LA, Eldridge SR, Elcombe CR (2003). Sub-chronic dietary toxicity of potassium perfluorooctanesulfonate in rats.. Toxicology.

[r37] Seacat AM, Thomford PJ, Hansen KJ, Olsen GW, Case MT, Butenhoff JL (2002). Subchronic toxicity studies on perfluorooctanesulfonate potassium salt in cynomolgus monkeys.. Toxicol Sci.

[r38] Starling AP, Engel SM, Whitworth KW, Richardson DB, Stuebe AM, Daniels JL (2014). Perfluoroalkyl substances and lipid concentrations in plasma during pregnancy among women in the Norwegian Mother and Child Cohort Study.. Environ Int.

[r39] Steenland K, Tinker S, Frisbee S, Ducatman A, Vaccarino V (2009). Association of perfluorooctanoic acid and perfluorooctane sulfonate with serum lipids among adults living near a chemical plant.. Am J Epidemiol.

[r40] Stein CR, Wolff MS, Calafat AM, Kato K, Engel SM (2012). Comparison of polyfluoroalkyl compound concentrations in maternal serum and amniotic fluid: a pilot study.. Reprod Toxicol.

[r41] Takacs ML, Abbott BD (2007). Activation of mouse and human peroxisome proliferator-activated receptors (α, β/δ, γ) by perfluorooctanoic acid and perfluorooctane sulfonate.. Toxicol Sci.

[r42] Thibodeaux JR, Hanson RG, Rogers JM, Grey BE, Barbee BD, Richards JH (2003). Exposure to perfluorooctane sulfonate during pregnancy in rat and mouse. I: maternal and prenatal evaluations.. Toxicol Sci.

[r43] United Nations Environment Programme. (2007). POPRC3: Development of Risk Management Evaluation. UNEP/POPS/POPRC.3/20.. http://chm.pops.int/Portals/0/Repository/poprc3/UNEP-POPS-POPRC.3-POPRC-3-5.English.PDF.

[r44] Vanden Heuvel JP, Thompson JT, Frame SR, Gillies PJ (2006). Differential activation of nuclear receptors by perfluorinated fatty acid analogs and natural fatty acids: a comparison of human, mouse, and rat peroxisome proliferator-activated receptor-α, -β, and -γ. liver X receptor-β, and retinoid X receptor-α.. Toxicol Sci.

[r45] Washino N Saijo Y Sasaki S Kato S Ban S Konishi K 2009 Correlations between prenatal exposure to perfluorinated chemicals and reduced fetal growth. Environ Health Perspect 117 660 667; 10.1289/ehp.11681 19440508 PMC2679613

[r46] Winquist A Steenland K 2014 Modeled PFOA exposure and coronary artery disease, hypertension, and high cholesterol in community and worker cohorts. Environ Health Perspect 122 1299 1305; 10.1289/ehp.1307943 25260175 PMC4256699

