# Supplementary material for: The Association of Prenatal Exposure to Perfluorinated Chemicals with Maternal Essential and Long-Chain Polyunsaturated Fatty Acids during Pregnancy and the Birth Weight of Their Offspring: The Hokkaido Study
Source: Environ Health Perspect. 2015 Apr 3;123(10):1038–45. doi: 10.1289/ehp.1408834 (PMC4590753; doi:10.1289/ehp.1408834)
Supplement: (139 KB) PDF [file ehp.1408834.s001.acco.pdf]

Environ Health Perspect
doi: 10.1289/ehp.1408834

Note to Readers: *EHP* strives to ensure that all journal content is accessible to all readers. However, some figures and Supplemental Material published in *EHP* articles may not conform to 508 standards due to the complexity of the information being presented. If you need assistance accessing journal content, please contact ehp508@niehs.nih.gov. Our staff will work with you to assess and meet your accessibility needs within 3 working days.

Supplemental Material

The Association of Prenatal Exposure to Perfluorinated Chemicals with Maternal Essential and Long-Chain Polyunsaturated Fatty Acids during Pregnancy and the Birth Weight of their Offspring: The Hokkaido Study

Reiko Kishi, Tamie Nakajima, Houman Goudarzi, Sachiko Kobayashi, Seiko Sasaki, Emiko Okada, Chihiro Miyashita, Sachiko Itoh, Atsuko Araki, Tamiko Ikeno, Yusuke Iwasaki, and Hiroyuki Nakazawa

Table of Contents

Table S1. The adjusted[a] least square means (LSMs) for the lipid levels by quartiles[b] of PFOS during pregnancy (n=306).

Table S1. The adjusted[a] least square means (LSMs) for the lipid levels by quartiles[b] of PFOS during pregnancy (n=306).

**Exposure**	**N**	**Crude LSMs (95% CI)**	**Adjusted LSMs (95% CI)**
TG (mg/dL)			
Quartile 1	77	Reference	Reference
Quartile 2	72	-13.1 (-26.6, 0.3)	-10.1 (-25.1, 4.7)
Quartile 3	80	-25.5 (-37.9, -13.1)	-19.5 (-33.2, -5.8)
Quartile 4	77	-22.2 (-34.9, -9.5)	-16.1 (-30.5, -1.7)
p for trend		**<.001**	**<.003**
Palmitic acid (μg/mL)			
Quartile 1	77	Reference	Reference
Quartile 2	72	-312.2 (-560.2, -64.3)	-246.6 (-544.3, 51.0)
Quartile 3	80	-505.6 (-738.2, -272.9)	-441.2 (-716.0, -166.4)
Quartile 4	77	-470.1 (-706.2, -233.9)	-422.1 (-706.9, -13.7.3)
p for trend		**<.001**	**<.001**
Palmitoleic acid (μg/mL)			
Quartile 1	77	Reference	Reference
Quartile 2	72	-26.2 (-47.3, -5.2)	-19.0 (-43.4, 5.3)
Quartile 3	80	-38.4 (-58.2, -18.6)	-31.2 (-53.7, -8.7)
Quartile 4	77	-40.7 (-60.5, -20.9)	-32.4 (-55.5, -9.4)
p for trend		**<.001**	**<.001**
Stearic acid (μg/mL)			
Quartile 1	77	Reference	Reference
Quartile 2	72	-19.4 (-76.5, 37.5)	-9.7 (78.7, 59.3)
Quartile 3	80	-6.6 (-62.8, 49.5)	-7.4 (-73.3, 58.5)
Quartile 4	77	8.7 (-48.8, 66.2)	-4.7 (-73.6, 64.1)
p for trend		0.671	0.900
Oleic acid (μg/mL)			
Quartile 1	77	Reference	Reference
Quartile 2	72	-188.4 (-348.5, -28.3)	-139.2 (-326.3, 47.8)
Quartile 3	80	-310.0 (-459.8, -160.3)	-247.4 (-420.3, -74.6)
Quartile 4	77	-276.6 (-429.3, -123.9)	-217.5 (-398.0, -37.0)
p for trend		**<.001**	**0.002**
Linoleic acid (μg/mL)			
Quartile 1	77	Reference	Reference
Quartile 2	72	-55.7 (-253.1, 141.6)	-73.0 (-333.7, 187.5)
Quartile 3	80	-306.9 (-471.8, -141.9)	-347.8 (-556.1, -129.4)
Quartile 4	77	-331.4 (-494.9, -168.0)	-373.6 (-593.4, -153.7)
p for trend		**<.001**	**<0.001**
α-Linolenic acid (μg/mL)			
Quartile 1	77	Reference	Reference
Quartile 2	72	-1.4 (-4.4, 1.5)	-1.7 (-5.7, 2.2)
Quartile 3	80	-4.1 (-6.8, -1.5)	-4.7 (-8.2,-1.2)
Quartile 4	77	-4.6 (-7.2, -2.0)	-5.2 (-8.6, -1.7)
p for trend		**<.001**	**<.001**
Arachidonic acid (μg/mL)			
Quartile 1	77	Reference	Reference
Quartile 2	72	1.7 (-15.5, 19.0)	-0.6 (-24.1, 22.9)
Quartile 3	80	-12.9 (-28.0, 2.0)	-19.3 (-39.4, 0.7)
Quartile 4	77	-17.8 (-32.4, -3.1)	-24.9 (-44.9, -5.0)
p for trend		0.003	**<.001**

Exposure	N	Crude LSMs (95% CI)	Adjusted LSMs (95% CI)
EPA[c] (µg/mL)			
Quartile 1	77	Reference	Reference
Quartile 2	72	1.5 (-0.4, 3.6)	1.4 (-1.1, 3.9)
Quartile 3	80	1.9 (-0.0, 4.0)	4.1 (-0.7, 4.0)
Quartile 4	77	1.3 (-0.5, 3.3)	0.9 (-1.4, 3.3)
p for trend		0.159	0.340
DHA[c] (µg/mL)			
Quartile 1	77	Reference	Reference
Quartile 2	72	4.0 (-3.0, 11.2)	3.5 (-6.7, 13.9)
Quartile 3	80	-0.4 (-6.7, 5.7)	-3.6 (-12.3, 5.0)
Quartile 4	77	-3.3 (-9.2, 2.5)	-6.4 (-15.0, 2.1)
p for trend		0.143	**<.03**
EFAs (µg/mL)			
Quartile 1	77	Reference	Reference
Quartile 2	72	-57.4 (-257.7, 142.8)	-75.2 (-339.8, 189.3)
Quartile 3	80	-311.4 (-478.9, -144)	-353.4 (-575.1, -131.6)
Quartile 4	77	-336.3 (-502.2, -170.3)	-379.5 (-602.8, -156.1)
p for trend		**<.001**	**<.001**
Omega 6 (µg/mL)			
Quartile 1	77	Reference	Reference
Quartile 2	72	-55.6 (-268.8, 157.4)	-75.2 (-357.3, 206.7)
Quartile 3	80	-322.2 (-500.9, -143.5)	-369.4 (-606.1, -132.7)
Quartile 4	77	-350.6 (-527.6, -173.7)	-399.7 (-637.9, -161.5)
p for trend		**<.001**	**<.001**
Omega 3[c] (µg/mL)			
Quartile 1	77	Reference	Reference
Quartile 2	72	3.9 (-5.8, 13.6)	3.0 (-9.9, 16.1)
Quartile 3	80	-1.0 (-9.9, 7.9)	-4.1 (-15.7, 7.4)
Quartile 4	77	-4.0 (-12.7, 4.6)	-7.3 (-19.0, 4.2)
p for trend		0.233	0.068

[a]Adjusted for maternal age, smoking and alcohol intake during pregnancy, annual household, parity, and blood sampling period (categorical). [b]Serum PFOS quartiles: quartile 1 (1.5-4 ng/mL), quartile 2 (4-5.6 ng/mL), quartile 3 (5.6-7.5 ng/mL), and quartile 4 (7.5-16.2 ng/mL). [c]For EPA, DHA and omega 3 FAs, adjusted model 1 and 2 also included fish intake.

After $\log_{10}$-transformation of PFOS and lipid levels (TG and fatty acids), PFOS levels were divided into four quartiles. Then, least square means (LSMs) and 95% confidence interval (CI) were calculated, and the LSMs and CIs were back transformed from $\log_{10}$ to normal values. *p* for trend were calculated by linear contrast.
